# The antidepressant-like activity of AC-5216, a ligand for 18KDa translocator protein (TSPO), in an animal model of diabetes mellitus

**DOI:** 10.1038/srep37345

**Published:** 2016-11-25

**Authors:** Zhi-Kun Qiu, Jia-Li He, Xu Liu, Guan-Hua Zhang, Jia Zeng, Hong Nie, Yong-Gang Shen, Ji-Sheng Chen

**Affiliations:** 1 Pharmaceutical Department of the First Affiliated Hospital of Guangdong Pharmaceutical University, Clinical Pharmacy Department of Guangdong Pharmaceutical University, Guangzhou, 510080, P.R. China; 2 College of Pharmacy, Jinan University, Guangzhou 510632, P.R. China; 3 Department of Endocrinology, Guangdong Provincial Hospital of Chinese Medicine, Guangzhou 510120, P.R. China; 4 Pharmacy Department of General Hospital of Chinese People’s Armed Police Forces, Beijing 100039, P.R. China; 5 Academy of Military Medical Sciences, Beijing 100850, P.R. China; 6 Neurosurgery Department of the Third Affiliated Hospital of Southern Medical University, Guangzhou 510060, P.R. China

## Abstract

Diabetes mellitus is a chronic disease that is associated with depression. Also, depression is common in adults with type 2 diabetes mellitus (T2DM). Translocator protein (18kDa) (TSPO) and allopregnanolone play an important role in the depression treatment. However, few studies have evaluated TSPO and allopregnanolone in the treatment of depression in T2DM. AC-5216, a ligand for TSPO, produces anxiolytic- and antidepressant-like effects in animal models. The present study aimed to explore antidepressant-like effects of AC-5216 on diabetic rats. Following the development of diabetic model induced by high fat diet (HFD) feeding and streptozotocin (STZ), AC-5216 (0.3 and 1 mg/kg, i.g.) elicited the antidepressant-like effects in behavioral tests while these activities were blocked by TSPO antagonist PK11195 (3 mg/kg, i.p.). The levels of allopregnanolone in the prefrontal cortex and hippocampus were increased by AC-5216 (0.3 and 1 mg/kg, i.g.), which was antagonized by PK11195 (3 mg/kg, i.p.). The increased plasma glucose (PG) and decreased insulin (INS) in HFD-STZ rats were reversed by AC-5216 (0.3 and 1 mg/kg, i.g.). This study indicates that the antidepressant-like effects of AC-5216 on HFD-STZ rats, suggesting that TSPO may represent a novel therapeutic target for depression in T2DM.

Diabetes mellitus is a metabolic disease that induces decreased quality and expectancy of life, as well as increased costs of care^1^. World Health Organization (WHO) has forecasted that about 300 million people will suffer from diabetes mellitus by 2025^2^. As compared to type 1 diabetes mellitus (T1DM), type 2 diabetes mellitus (T2DM) is much more prevalent. It comprises around 90% of people with diabetes mellitus that is caused by a combination of resistance to insulin action and an inadequate compensatory insulin secretory response^3^. The structural and neurophysiological changes in the central nervous system (CNS) are induced by diabetes that are associated with cognitive deficits and the psychiatric disorder, such as depression^4^,^5^.

Depression is a common co-morbid condition in T2DM and it has been estimated that people with T2DM are more likely to suffer from depression than general population^5^. Comorbid depression is also associated with increased risks of diabetic retinopathy, neuropathy, nephropathy, macrovascular complications, and sexual dysfunction^6^. Patients with depression had a 60% increased risk of developing T2DM^7^. The possibility is that people with elevated depressive symptoms are less attentive toward a healthy lifestyle, therefore increasing the risks of developing diabetes^8^. Furthermore, depression in T2DM is related to the diminished quality of life. The impact of depression on quality of life in various chronic diseases (arthritis, angina, asthma and diabetes) shows that quality of life is impaired in patients with diabetes and depression^9^.

Despite a variety of psychological and pharmacological treatments have been introduced in depression with T2DM, a large proportion of patients had not achieved satisfactory outcomes. One finding refers to the chronic dysregulations of serotonin (5-HT) activity leading to the pathogenesis of depression in T2DM^10^. Numerous evidences support that selective serotonin reuptake inhibitors (SSRIs) (e.g fluoxetine and sertraline) are effective in the therapy of depression in T2DM^10^,^11^. However, SSRIs have disadvantages, including delayed onset of action, limited response with residual symptoms as well as severe side effects, such as insomnia and agitation^12^. Based on the downsides, investigators need to search the novel pharmacological targets for depression in T2DM treatments.

One major role of neuroactive steroid (i.e., allopregnanolone) is neuroprotection in case of lesion, ischemia or peripheral neuropathies (i.e., diabetes)^13^. Allopregnanolone, a neuroactive steroid derived from progesterone, is synthesized within the nervous tissue, by means of specific enzymes^13^. Contrary to progesterone and its metabolite dihydroprogesterone, allopregnanolone is able to interact with γ-aminobutyric acid (GABA) A receptor that is associated with psychiatric disorders, such as schizophrenia, depression, anxiety, and impulsive aggression^14^,^15^,^16^. Lowered levels of allopregnanolone may lead to imbalance in excitatory neurotransmission that causes depressive symptoms^17^. Conversely, allopregnanolone exerts antidepressant-like effects in preclinical and clinical studies^16^,^18^. However, the relationship between allopregnanolone and depression in T2DM has not yet fully understood. Thus, it is reasonable to hypothesis that allopregnanolone may also play a significant role in the treatment of depression in T2DM.

Translocator protein 18kDa (TSPO), previously described as peripheral-type benzodiazepine receptor (PBR), represents the starting point and an important rate-limiting step in neurosteroidogenesis^19^. It is a five trans-membrane domain protein that is located mainly in the outer mitochondrial membrane in peripheral tissues and central nervous system (CNS). In the CNS, TSPO is basically located in the glial cells and mediates the translocation of cholesterol from outer to inner mitochondrial membrane^20^. The role of TSPO has received increased attention on the pathophysiology of stress response and stress-related disorders^21^. The alternation of TSPO expression (or function) is a promising therapeutic target for depression without benzodiazepine-like side effects^22^. In diabetic study, TSPO activation was effective in ameliorating the severity of diabetic neuropathy by means of increased levels of neuroactive steroid^23^. Consequently, TSPO ligands may be represented as a promising option on the remedy of neurological disorders. The therapeutic approach could be extremely interesting because it may avoid possible endocrine side effects exerted by systemic treatment of neuroactive steroids.

The TSPO ligand, such as AC-5216 (Emapunil, XBD173), has been shown to exert anxiolytic/antidepressant activity in rodents by neurosteroidogenesis^24^. In addition, the TSPO ligands reduced the weight gain and improved glucose tolerance to high-fat diet-induced obese rodents^23^. However, little is known about the importance of TSPO in the treatment of depression in T2DM.

In the present study, the development of diabetic model in rats was induced by high fat diet (HFD) feeding and streptozotocin (STZ). STZ is widely used in T2DM animal models preparation. It is a glucosamine-nitrosourea that acts by alkylating DNA and exposing cells to reactive oxygen species and nitric oxide^25^. STZ accumulates efficiently in pancreatic β-cells resulting in cell death that routinely deployed to induce experimental diabetes^26^. Following the development of HFD-STZ rats, we evaluated the pharmacological characteristics of AC-5216. The antidepressant-like effects were assessed by animal behavioral tests, including sucrose preference test (SPT), novelty-suppressed feeding test (NSFT), forced swimming test (FST) and open-field test (OFT). To further evaluate the role of TSPO in the treatment of depression in T2DM, we determined whether pharmacological effects of AC-5216 were antagonized by PK11195 (TSPO antagonist) in HFD-STZ rats. The animals were decapitated after the end of behavioral tests. The levels of allopregnanolone, plasma glucose (PG), insulin (INS), total cholesterol (TC), and triglyceride (TG) were assessed as well.

## Materials and Methods

### Drugs and administration

AC-5216 was purchased from Medchem Express (USA). PK11195, STZ, metformin (Met) and fluoxetine (Flu) were purchased from Sigma-Aldrich (USA). AC-5216 was prepared as a suspension in 0.5% tragacanth gum solution and administered to rats by gavage (i.g)^27^. The dose range of AC-5216 (0.1, 0.3, and 1 mg/kg, i.g.) was based on its antidepressant- and anxiolytic- like activities as described previously with minor adjustments^24^. PK11195, injected intraperitoneally (i.p.), was suspended in saline containing 2% DMSO and 0.8% Tween 80^24^. The dosage of PK11195 (1 and 3 mg/kg, i.p.) was based on published studies showing the inhibitory effect of PK11195 against AC-5216 and other TSPO ligands^24^,^28^,^29^. STZ dissolved freshly in citrate buffer (pH 4.5)^30^. With the control effects of blood glucose, Met is the mainstay treatment in the prevention of T2DM and associated comorbidities^31^. The lowered blood glucose induced by Met in HFD-STZ model mimics the situation of type 2 diabetes relevant to human condition. Consequently, Met (1.8 mg/kg, i.p), Flu (10.8 mg/kg, i.p) and the combination (Met+Flu, MF) were administered as positive control drugs in all behavioral tests respectively based on their antidepressant-like effects on the T2DM rodent model^30^.

### Animals and housing

Sprague-Dawley rats (male, 180 ± 20 g) were purchased from Beijing Vital Laboratory Animal Technology Company (Beijing, China). The animals were maintained in the standard conditions of controlled temperature (23 ± 1 °C), humidity (45%), and lighting (from 06:00 to 18:00). The rats were housed in a 12-h light/dark cycle starting at least 5 days before the experiments with access to food and water freely available. The total number of animals was 100 (ten in each group). All methods were carried out in accordance with National Institute of Health Guide for the care and Use of Laboratory Animals (NIH Publications No. 80–23, revised 1996). The experimental procedures were approved by the institutional committee of Academy of Military Medical Sciences on animal care and use. All efforts were made to minimize animal suffering and reduce the number of animals used in the experiments.

### Development of the HFD-STZ rats

The development of the HFD-STZ rats mimics the situation T2DM in human^30^. Development of HFD-STZ rats was performed by basing the design on previous studies with minor adjustments^10^,^30^. After the accommodation to new environment, rats were divided randomly into two groups: the control group (HFD-STZ (−), n = 10) was fed a normal chow diet, whereas the diabetic group (HFD-STZ (+), n = 90) was fed a HFD (59% fat, 26% protein and 20% carbohydrate, as a percentage of total kcal) for two weeks. After the duration of dietary manipulation, the diabetic groups were fasted for 16 h followed by a dose of STZ (40 mg/kg, i.p) for two consecutive days. While the control group was given vehicle citrate buffer (pH 4.5) in a volume of 2 mL/kg, i.p, respectively. PG was determined by a single touch glucometer (OneTouch Ultra 2; LifeScan, High Wycombe, UK). The rat with the non-fasting PG ≥ 300 mg/dl was considered diabetic and selected for further evaluation of the feasible preparation of HFD-STZ rats^30^. After that, oral glucose tolerance test (OGTT) was performed based on the previous study^10^. Animals were fasted for 16 h and given glucose at a dose of 2 g/kg, i.p. PG was determined before and after 15, 30, 60, 90 and 120 min of the glucose application. In addition, during the development of HFD-STZ rats, the body weight (BW) was recorded for all of the rats. We found that within the period of HFD-STZ rat spreparation, no significant difference of BW between control group and diabetic group (data not shown). However, OGTT showed severely impaired clearance of acute increase of PG during 120 min monitoring process: the levels of PG in HFD-STZ rats were significantly higher than that of control rats at all of the time points (data not shown).

### Behavioral assessments

**Figure 1:**
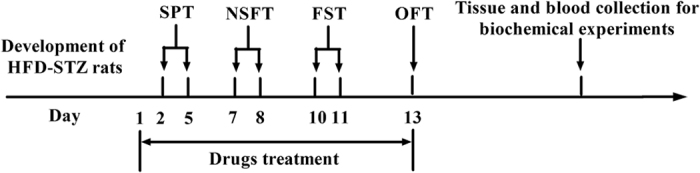
Treatment schedule and order of behavioral tests for the development of HFD-STZ rats.

Two days after the development of HFD-STZ rats, behavioral experiments were started. The sucrose preference test (SPT) (from day 2 to 5), novelty-suppressed feeding test (NSFT) (from day 7 to 8), forced swimming test (FST) (from day 10 to 11), and open-field test (OFT) (day 13) were conducted. To evaluate the effects of repeated treatments on behavior, Met (1.8 mg/kg, i.p), Flu (10.8 mg/kg, i.p), MF, AC-5216 (0.1, 0.3, and 1 mg/kg, i.g.) and PK11195 (1 and 3 mg/kg, i.p.) were given once per day from day 1 to 13. Met, Flu, MF and AC-5216 were given 1 h and PK11195 was given 30 min before the behavioral tests, respectively. The control rats (HFD-STZ (-) were given vehicle 0.5% tragacanth gum solution. The outline of treatment schedule design and behavioral tests is shown in Fig. 1.

### Sucrose preference test (SPT)

SPT is widely employed to measure major depression in human^32^. The test was performed as described previously^33^. Rats were placed in individual cages to train to consume 1% (w/v) sucrose solution for 48 h without food and water supply. Following 16 h water deprivation, one bottle of 1% sucrose solution was replaced by water. The SPT was performed for 1 h. During the test, rats could select between two preweighed bottles, one with 1% sucrose solution and the other with tap water. The sucrose preference was calculated as sucrose intake/(sucrose intake + water intake) × 100%.

### Novelty-suppressed feeding test (NSFT)

The NSFT provides a sensitive and reliable measure of depression and motivation level in animals which mimics the situation in human^34^. The test was performed according to the literature with minor modification^35^. Briefly, after fasting for 24 h, rats were placed in the corner of the plastic box (76 × 76 × 46 cm) with several pellets placed in the center. The latency to begin eating within 5 min was recorded (defined as rat chewing or biting the pellet, instead of merely sniffing or toying with it). Moreover, home-cage food consumption in 5 min was immediately evaluated to assess effects of drugs on feeding drive.

### Forced swimming test (FST)

FST is a well-established measurement for evaluation the effects of antidepressants. The antidepressant-like effects are indicated by decreased duration of immobility. The test is highly reliable to predict the validity of antidepressants^29^. The procedure comprised two sessions (the pretest and the test) with identical apparatus and conditions (height 40 cm, diameter 20 cm, containing 25 cm of water maintained at 25 °C). During the pretest session, rats were forced to swim for 15 min. After 24 h, rats were placed in the same apparatus for 5 min and the session was designated as a test session. The duration of immobility during 5 min was measured.

### Open-field test (OFT)

In order to rule out whether non-specific locomotor effects of AC-5216 on antidepressant-like activity, the locomotor activity was assessed. The test was performed as described previously^35^. Rats were placed in the corner of plastic box (76 × 76 × 46 cm) for 5-min acclimation, then the number of crossings (squares crossed with all paws) and rears (raising the fore paws) and fecal pellets was recorded in the next 5 min session. The square arena was cleaned with a water-alcohol (10%) solution and dried after each test in order to hide animal cues and prevent the odor influence from the previous animals.

### Blood collection and levels of PG, INS, TC, and TG measurement

To evaluate effects of AC-5216 on levels of PG, INS, TC, and TG, which are important parameters in diabetic animal models^30^,^36^, the blood was collected and the levels of parameters above were determined followed by the final behavioral test as the previous study with minor adjustments^36^. The single touch glucometer (OneTouch Ultra 2; LifeScan, High Wycombe, UK) was used to determine the levels of PG collected from the tail vein of rats. The blood sample was centrifuged (2000 g, 25 min) at 4 °C, and supernatants were collected. All samples were maintained at −80 °C until further use. The samples were assayed and quantified by Enzyme Immunoassay kits, respectively (INS (Millipore, USA), TC (Cell Biolabs, USA), and TG (Abcam, USA)). Six samples in each group were used to determine optical density (OD) values, at 450 nm in enzyme-linked immunosorbent assay (ELISA) plate reader and used for statistical analyzes.

### Allopregnanolone determination

Altered levels of allopregnanolone have been implicated as one of the possible contributors to the development of depression^17^. The levels of allopregnanolone in the antidepressant-like activity of AC-5216 were also evaluated by ELISA. The brain and blood preparation for ELISA was based on a literature^37^. Animals were sacrificed by decapitation at the end of OFT in 24 h after the last drugs treatment. The brains were removed, rinsed of blood, and carefully dissected to remove the prefrontal cortex and hippocampus. Both brain regions, involved in emotional processing, fear conditioning and explicit memory, play a significant role in depression^38^,^39^.

The brain regions were extracted by 1 mL extraction buffer per 100 mg tissue and then homogenized in the ice-cold lysis buffer containing 137 mM NaCl, 1% NP40, 10% glycerol, 20 mM Tris-HCl (pH 8.0), 1 μg/mL leupeptin, 1 mM PMSF 10 μg/mL aprotinin, and 0.5 mM sodium vanadate. The tissue homogenate solutions were centrifuged at 13,000 g for 30 min at 4 °C, and then the supernatants were collected. For the measurement of serum allopregnanolone, the blood samples were centrifuged (2000 g, 25 min) at 4 °C, and the supernatants were collected. All samples were maintained at −80 °C until further use. Allopregnanolone was quantified by Enzyme Immunoassay kit (Raybiotech, USA) that was specific for the measurement of allopregnanolone^40^. Six samples in each group were used to determine OD values at 450 nm in ELISA plate reader and used for statistical analyzes.

### Statistical analysis

Unless otherwise specified, statistical analysis was conducted by GraphPad Prism 5.0 (version 2.0; GraphPad Software Inc., San Diego, CA). All data are presented as mean ± standard error of measurement (S.E.M). The statistical significance of experimental observations was determined by one-way analysis of variance (ANOVA) or two-way ANOVA followed by Bonferroni’s multiple comparison tests, as indicated in the results section. For all tests, level of statistical significance was set at *p *< 0.05.

## Results

### The effects of AC-5216 on HFD-STZ rats in SPT

**Figure 2:**
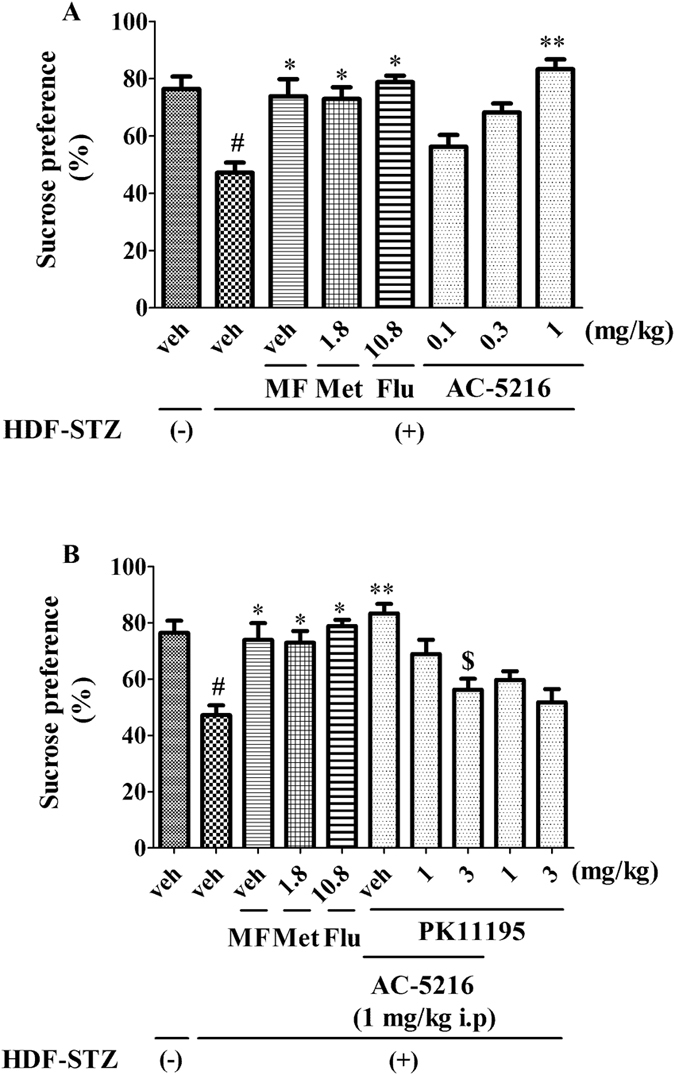
The antidepressant-like effects of AC-5216 on HFD-STZ rats in SPT. The sucrose preference was increased by AC-5216 (**A**) and this effect was reversed by PK11195 (**B**) ^#^*p* < 0.05 vs. vehicle-treated HFD-STZ (−) group; *p < 0.05,**p < 0.01 vs. vehicle-treated HFD-STZ (+) group; ^$^*p* < 0.05 vs. AC-5216 (1 mg/kg, i.p.) group (n = 10).

The antidepressant-like effects of AC-5216 on HFD-STZ rats in SPT were shown in Fig. 2. Sucrose preference was significantly decreased in HFD-STZ rats. Similar to Met (1.8 mg/kg, i.p), Flu (10.8 mg/kg, i.p) and MF, AC-5216 (0.3 and 1 mg/kg, i.g) produced antidepressant-like effects, as evidenced by the increase of sucrose preference (one-way ANOVA, F (7, 72) = 9.245, *p *< 0.05; Fig. 2A). However, this activity (AC-5216, 1 mg/kg, i.g.) was antagonized by PK11195 (3 mg/kg, i.p.) (two-way ANOVA, F (9, 90) = 8.798, *p *< 0.05; Fig. 2B), indicating that the antidepressant-like effects of AC-5216 in SPT were mediated by TSPO.

### The effects of AC-5216 on HFD-STZ rats in NSFT

**Figure 3:**
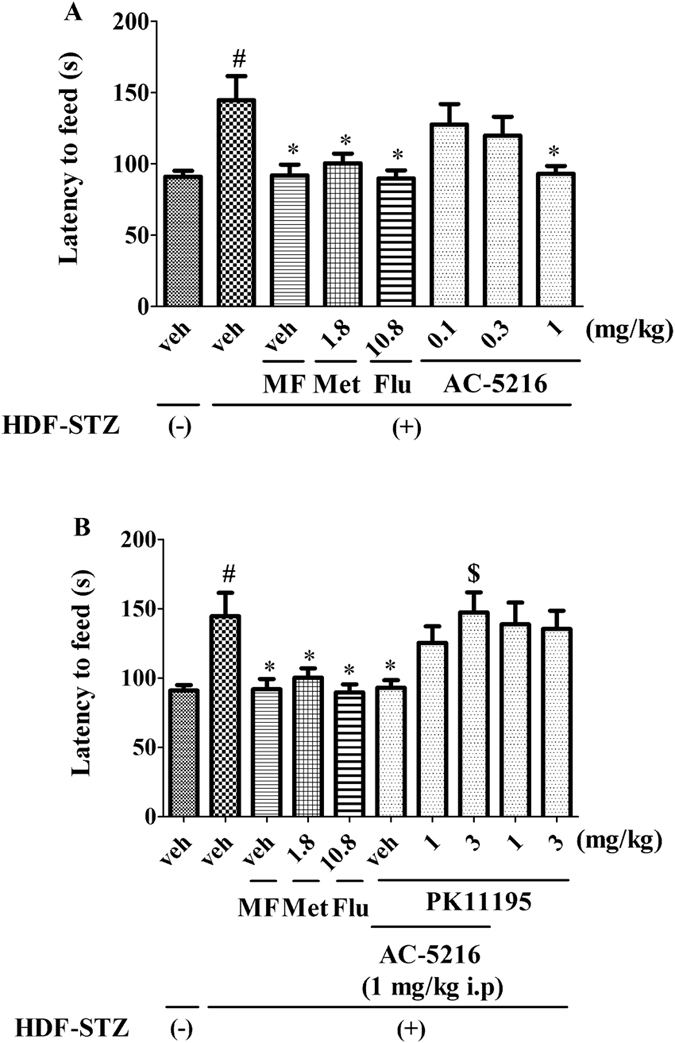
The antidepressant-like effects of AC-5216 on HFD-STZ rats in NSFT. The latency to feed was decreased by AC-5216 (**A**) and these effects were reversed by PK11195 (**B**) ^#^*p* < 0.05 vs. vehicle-treated HFD-STZ (−) group; *p < 0.05 vs.vehicle-treated HFD-STZ (+) group; ^$^*p* < 0.05 vs. AC-5216 (1 mg/kg, i.p.) group (n = 10).

As shown in Fig. 3, the latency to feed was increased significantly in HFD-STZ rats. Consistent with Met (1.8 mg/kg, i.p), Flu (10.8 mg/kg, i.p) and MF, AC-5216 (1 mg/kg, i.g) produced the antidepressant-like effects, as evidenced by the decrease of latency to feed (one-way ANOVA, F (7, 72) = 4.072, *p *< 0.05; Fig. 3A). However, the effects (AC-5216, 1 mg/kg, i.g.) were blocked by PK11195 (3 mg/kg, i.p.) (two-way ANOVA, F (9, 90) = 4.914, *p *< 0.05; Fig. 3B). Moreover, no differences of in home-cage food consumption were observed among the groups (data not shown). These results indicated that the antidepressant-like effects of AC-5216 in NSFT were mediated by TSPO.

### The effects of AC-5216 on HFD-STZ rats in FST

**Figure 4:**
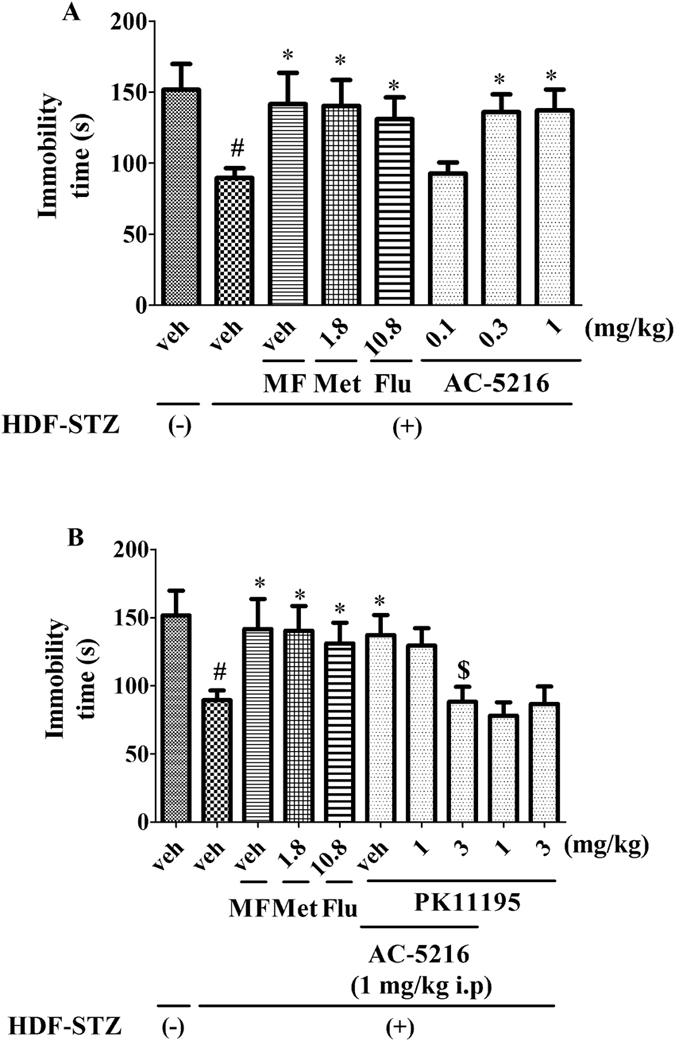
The antidepressant-like effects of AC-5216 on HFD-STZ rats in FST. The immobility time was decreased by AC-5216 (**A**). The antidepressant-like effects of AC-5216 were antagonized by PK11195 in the immobility time (**B**). ^#^*p *< 0.05 vs. vehicle-treated HFD-STZ (−); *p < 0.05 vs. vehicle-treated HFD-STZ (+) group; ^$^*p *< 0.05 vs. AC-5216 (1 mg/kg, i.p.) group (n = 10).

The antidepressant-like effects of AC-5216 on HFD-STZ rats in FST were shown in Fig. 4. The immobility time was increased significantly in HFD-STZ rats. Similar to Met (1.8 mg/kg, i.p), Flu (10.8 mg/kg, i.p) and MF, AC-5216 (0.3 and 1 mg/kg, i.g.) exerted the antidepressant-like effects, as evidenced by the decreased immobility time (one-way ANOVA, F (7, 72) = 2.328, *p *< 0.05; Fig. 4A). However, the activities of AC-5216 (1 mg/kg, i.g.) was antagonized by PK11195 (3 mg/kg, i.p.) (two-way ANOVA, F (9, 90) = 3.621, *p *< 0.05; Fig. 4B), indicating that the antidepressant-like effects of AC-5216 in FST were mediated by TSPO.

### The effects of AC-5216 on HFD-STZ rats in OFT

**Figure 5:**
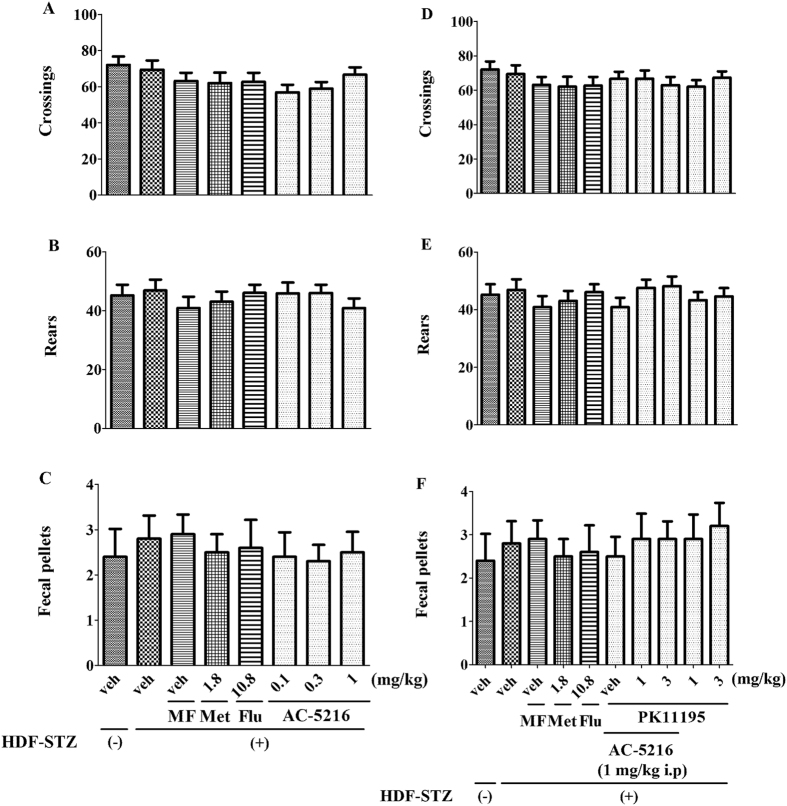
Effects of treatments on locomotor activity. None of the treatments altered the number of line crossings (**A**,**D**), rears (**B**,**E**), and fecal pallets (**C**,**F**) in OFT (n = 10).

The OFT is evaluated whether locomotor activity is affected by various treatments in rats. The effects of treatments on locomotor activity were shown in Fig. 5. One-way ANOVA analysis revealed that similar to the model group, the number of crossings (F (7, 72) = 1.213, *p *> 0.05; Fig. 5A), rears (F (7, 72) = 0.5026, *p *> 0.05; Fig. 5B) and fecal pallets (F (7, 72) = 0.1707, *p *> 0.05; Fig. 5C) was not affected by Met, Flu, MF and AC-5216. Also, two-way ANOVA analysis revealed that PK11195 had no effects on the crossings (F (9, 90) = 0.5635, *p *> 0.05; Fig. 5D), rears (F (9, 90) = 0.6367, *p *> 0.05; Fig. 5E) and fecal pallets (F (9, 90) = 0.2325, *p *> 0.05; Fig. 5F). These results indicated that the antidepressant-like effects of AC-5216 were not affected by locomotor activity in HFD-STZ rats.

### The effects of AC-5216 on levels of PG, TC, TG, and INS in HFD-STZ rats

**Figure 6:**
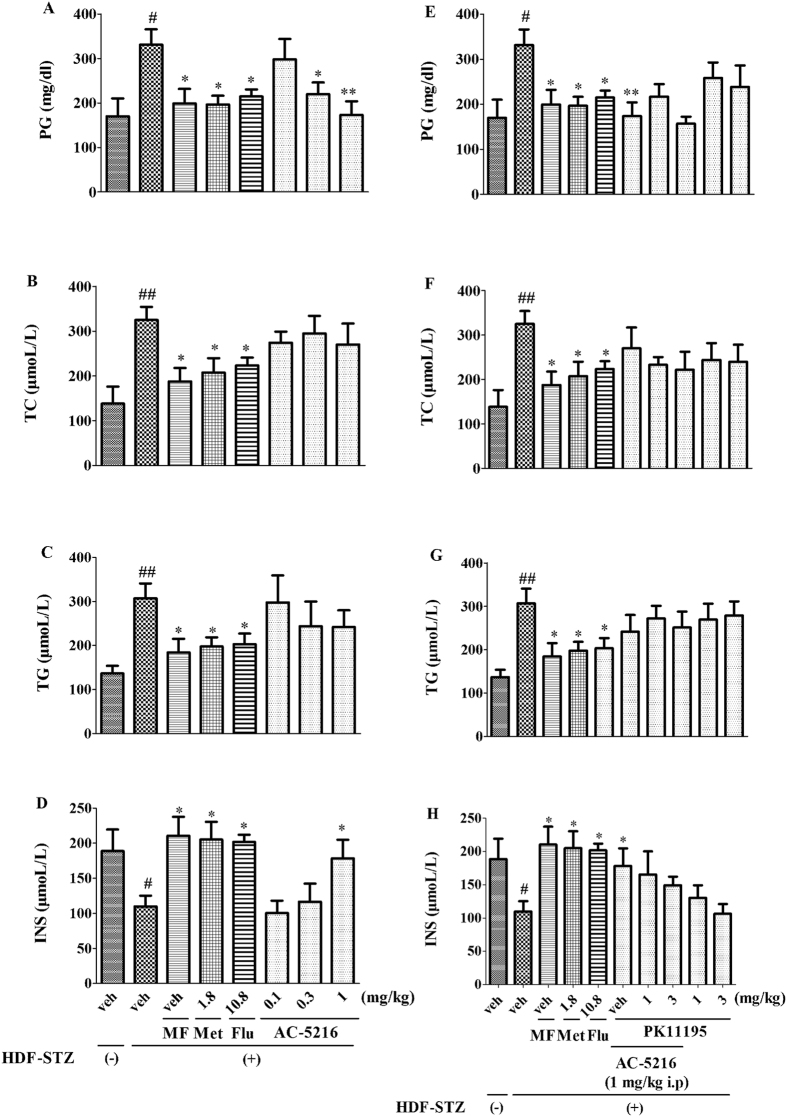
The effects of AC-5216 on PG, TC, TG, and INS in HFD-STZ rats. The increase of PG (**A**) and the decrease of INS (**D**) were reversed by AC-5216, which did not affect the levels of TC (**B**) and TG (**C**) Moreover, PG (**E**) TC (**F**) TG (**G**) and INS (**H**) in HFD-STZ rats were not affected by PK11195. ^#^*p* < 0.05, ^##^*p *< 0.01 vs. vehicle-treated HFD-STZ (−) group; *p < 0.05, **p < 0.01 vs. vehicle-treated HFD-STZ (+) group (n = 6).

The effects of AC-5216 on the levels of PG, TC, TG, and INS in HFD-STZ rats were shown in Fig. 6. One-way ANOVA analysis revealed that the levels of PG (F (7, 40) = 3.320, *p *< 0.05; Fig. 6A), TC (F (7, 40) = 3.426, *p *< 0.05; Fig. 6B), and TG (F (7, 40) = 2.258, *p *< 0.05; Fig. 6C) were increased significantly while the INS (F (7, 40) = 4.065, *p *< 0.05; Fig. 6D) was markedly decreased in HFD-STZ rats. Similar to Met (1.8 mg/kg, i.p), Flu (10.8 mg/kg, i.p) and MF, the increase of PG and the decrease of INS were reversed by AC-5216 (0.3 and 1 mg/kg, i.g for PG and 1 mg/kg, i.g for INS, respectively). Both TC and TG were not significantly affected by AC-5216. Moreover, two-way ANOVA analysis revealed that the levels of PG (F (9, 50) = 2.655, *p *< 0.05; Fig. 6E), TC (F (9, 50) = 2.081, *p *< 0.05; Fig. 6F), TG (F (9, 50) = 2.889, *p *< 0.05; Fig. 6G), and INS (F (9, 50) = 2.874, *p *< 0.05; Fig. 6H) in HFD-STZ rats were not affected by PK11195. These results indicated that AC-5216 induced the increase of PG and the decrease of INS.

### The effects of AC-5216 on allopregnanolone in HFD-STZ rats

**Figure 7:**
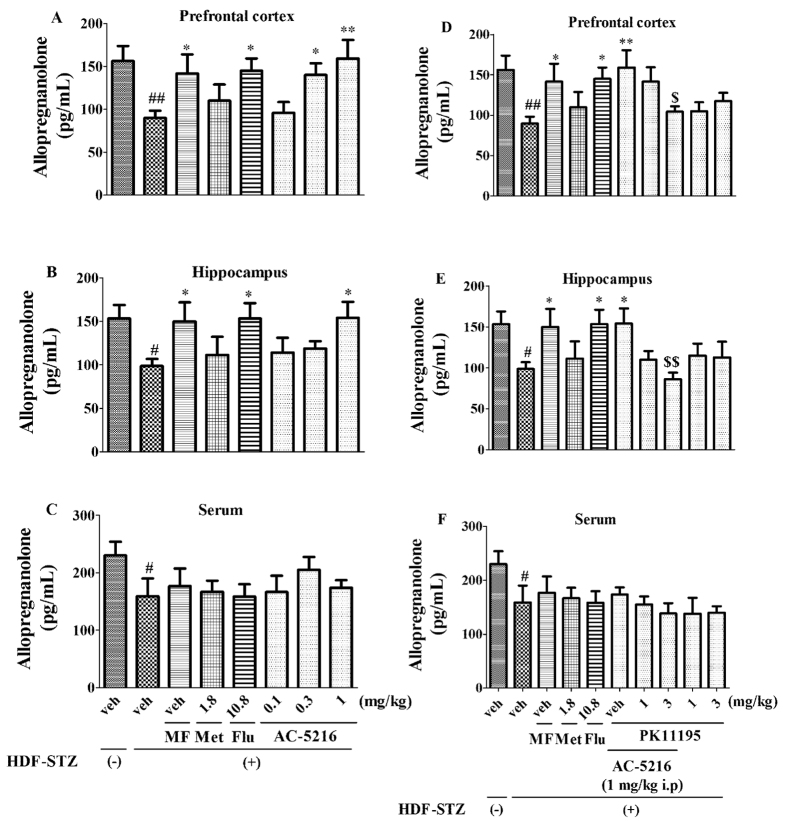
The effects of treatment on allopregnanolone in HFD-STZ rats. The decreased levels allopregnanolone were reversed by AC-5216 in the prefrontal cortex (**A**) and hippocampus (**B**) but not in serum (**C**). However, the effects of AC-5216 were totally blocked by PK11195 in the prefrontal cortex (**D**) and hippocampus (**E**), but not in serum (**F**). ^#^*p *< 0.05, ^##^*p *< 0.01 vs. vehicle-treated HFD-STZ (−) group; *p < 0.05, **p < 0.01 vs. vehicle-treated HFD-STZ (+) group; ^$^*p *< 0.05, ^$$^*p *< 0.01 vs. AC-5216 (1 mg/kg, i.p.) group (n = 6).

The effects of AC-5216 on allopregnanolone in HFD-STZ rats were illustrated in Fig. 7. One-way ANOVA analysis revealed that the levels of allopregnanolone were decreased significantly in the prefrontal cortex (F (7, 40) = 2.654, *p *< 0.05; Fig. 7A), hippocampus (F (7, 40) = 1.922, *p *< 0.05; Fig. 7B), and serum (F (7, 40) = 1.059, *p *< 0.05; Fig. 7C) in HFD-STZ rats. Similar to Flu (10.8 mg/kg, i.p) and MF, the effects were reversed by AC-5216 in the prefrontal cortex (0.3 and 1 mg/kg, i.g. for prefrontal cortex, Fig. 7A) and hippocampus (1 mg/kg, i.g. for hippocampus, Fig. 7B), but not in serum (Fig. 7C). However, two-way ANOVA analysis revealed that the effects of AC-5216 were totally blocked by PK11195 (3 mg/kg, i.p.) in the prefrontal cortex (F (9, 50) = 2.418, Fig. 7D) and hippocampus (F (9, 50) = 2.509, Fig. 7E) without in serum (F (9, 50) = 1.445, Fig. 7F). The results indicated that the antidepressant-like effects of AC-5216 in HFD-STZ rats were associated with allopregnanolone biosynthesis in the prefrontal cortex and hippocampus, which was mediated by TSPO.

## Discussion

This study was initiated with the objective of evaluating the role of TSPO and allopregnanolone biosynthesis in the treatment of depression in T2DM. Antidepressant-like pharmacological profile of AC-5216 was determined by animal behavioral tests. Based on the data above, the antidepressant-like activities were produced by AC-5216 in HFD-STZ rats and these effects were antagonized by TSPO antagonist PK11195 without affecting the locomotor activity. Also, the decreased allopregnanolone synthesis in the prefrontal cortex and hippocampus was reversed by AC-5216 while these effects were also antagonized by PK11195. It is indicated that the antidepressant-like effects of AC-5216 in HFD-STZ rats were mediated by TSPO and allopregnanolone biosynthesis.

The diabetic animal model (HFD-STZ rats) is typical, including either irreversible or reversible (insulin resistance) destruction of β-pancreatic cells, which is widely utilized in the T2DM study^25^. The model simulates the human syndrome of depression in DM and is identified as suitable for testing anti-diabetic agents for the treatment of T2DM^26^. The alternations of PG, INS, TC, and TG are important parameters to determine T2DM^30^,^36^. Comparing to the control group, PG (Fig. 6A), TC (Fig. 6B), and TG (Fig. 6C) were increased while INS (Fig. 6D) was decreased that the animals induced by HFD-STZ administration. These similar alternations in diabetic rodents were consistent with the previous studies demonstrating that the changes resembled the situation of patients with T2DM^26^,^30^,^41^.

Depression is prevalent in the patients with diabetes. The risks of elevated incident depression are increased in individuals with T2DM compared with those in healthy subjects^5^. Consequently, following the exposure to HFD-STZ treatment, depressive-like behavior was evaluated in rats. Our data indicated that depressive-like behavior was elicited by diabetic procedure, as evidenced by sucrose preference was significantly decreased in SPT (Fig. 2), the latency to feed and the immobility time was increased markedly in NSFT (Fig. 3) and FST (Fig. 4), respectively. All of these parameters are represented as depressive-like symptoms in rodents^33^. Similarly, clinical reports have indicated that depression is linked to hyperglycemia^42^. Depressed adults with T2DM have poor control of glycemia as compared to those without mood disorder^43^. An explanation for relationship between depression and T2DM stems from the study results indicating that blood glucose is itself a potent regulator for mood states^44^. In particular, hypoglycemia or severe hyperglycemia is able to induce negative emotional states in patients with diabetes^43^. Other studies has reported that the depressive mood is positively related to the presence of diabetic complications. It has been reported that prevalence of depression is higher among T2DM subjects with retinopathy, neuropathy, nephropathy and peripheral vascular disease (PVD)^45^,^46^. An explanation for the association could be the increase in the burden of disease due to complications which can lead to depressive-like symptoms^2^.

Numerous studies have reported that TSPO plays a significant role in the therapy of depression and symptoms of depression could be reversed by TSPO ligands (e.g AC-5216, YL-IPA08)^24^,^27^. However, the importance of TSPO in the remedy of depression in T2DM is still unknown. To evaluate this, several behavioral tests as measures of depression were performed after development of HFD-STZ rats. Interestingly, similar to traditional antidepressant (Flu)^47^, Met (1.8 mg/kg, i.p) also had antidepressant-like effects on HFD-STZ rodents. These findings were supported by the clinical research that Met produced antidepressant effects through improvement of cognitive function among depressed patients with diabetes mellitus^48^. The results of the present and previous studies raise the possibility that supplementary administration of antidiabetic medications may enhance the recovery of depression, comorbid with T2DM, through improvements in cognitive performance. Consistent with Met (1.8 mg/kg, i.p), Flu (10.8 mg/kg, i.p) and MF, AC-5216 produced the antidepressant-like effects in SPT (Fig. 2) (reversed the decreased sucrose preference (Fig. 2A)), in NSFT (Fig. 3) (reversed the increased latency to feed (Fig. 3A)), in FST (Fig. 4) (reversed the decreased immobility time (Fig. 4A)). The similarly effective doses (0.3 and 1 mg/kg, i.g) were consistent among the behavioral tests and in line with anxiolytic-like effects of AC-5216 in the Vogel conflict test and in the social interaction test^24^,^49^. Also, our previous studies showed that the behavioral deficits in an animal model of post-traumatic stress disorder (PTSD) were attenuated by AC-5216^37^,^50^. All the anxiolytic- and anti-PTSD- like effects were at similar doses, which supported our present study. The data above (Figs 2A, 3A, 4A) had implicated that antidepressant-like activity of AC-5216 in HFD-STZ rats was mediated by TSPO on the basis that AC-5216 had high binding affinity on TSPO.

To further evaluate the significance of TSPO on depression in T2DM, PK11195 was applied in the present study. PK11195 is an isoquinoline carboxamide which binds selectively to TSPO and widely utilized in the TSPO antagonism study^24^,^29^. The data showed that the antidepressant-like activities of AC-5216 in HFD-STZ rats was antagonized by PK11195 at the dose of 3 mg/kg i.p (Figs 2B, 3B, 4B). All these results were in agreement with previous studies showing the antidepressant-, anxiolytic-, and anti-PTSD- like effects of AC-5216 were blocked by PK11195^24^,^29^,^37^. Taking together, these demonstrate that TSPO plays a significant role in the treatment of depression in T2DM.

We also found that the antidepressant-like effects of AC-5216 were not significantly influenced by locomotor activity (Fig. 5), including crossings (Fig. 5A), rears (Fig. 5B) and fecal pellets (Fig. 5C) in HFD-STZ rats. The findings were consistent with the studies that anxiolytic-, anti-PTSD-, and anti-panic- like effects of AC-5216 were also not mediated by affecting the locomotor activity^22^,^49^,^50^. Our results were also consistent with other reports that TSPO selective ligands with subnanomolar affinity for TSPO such as indolylglyoxylamides, stimulated the steroid biosynthesis and exerted the anxiolytic activities without affecting the locomotor activity in rats^51^,^52^. Also, the locomotor activity in HFD-STZ rats was not affected by PK11195 (Fig. 5D–F). The data indicated that TSPO is a potential treatment target for depression in T2DM without affecting locomotor activity. In addition, our data were supported by a previous study showing that TSPO is a promising target for treating neurological disorders without benzodiazepine-like side effects, such as emotional and somatic withdrawal symptoms^53^.

Based on the data of behavioral tests indicating that the antidepressant-like effects of AC-5216 were mediated by TSPO in HFD-STZ rats, more investigation involved the significance of neurosteroid biosynthesis and pharmacological mechanism of AC-5216 was needed. Neurosteroids serve as neuromodulators at neurotransmitter receptors, (e.g acetylcholine and glutamate receptors), and also affect emotion, memory, learning as well as stress responses^54^,^55^. The development and maintenance of stress-associated depression might be ascribed to the altered steroid production^20^,^56^. Further, neurosteroids have been reported to exert the protective effects in animal models that mimic a variety of pathogenic aspects of brain dysfunction, including Alzheimer’s disease, traumatic brain injury, and stroke^56^,^57^. In diabetic study, levels of neuroactive steroid were decreased in CNS of STZ-treated rats^23^. Consistent with this finding, our findings showed that levels of allopregnanolone were decreased significantly in the prefrontal cortex (Fig. 7A), hippocampus (Fig. 7B), and serum (Fig. 7C) in HFD-STZ rats. More studies had shown that depression was closely associated with the neurosteroids biosynthesis (e.g allopregnanolone). For instance, decreased allopregnanolone in peripheral blood or cerebrospinal fluid (CSF) was found to relevant to major depression, anxiety disorders, premenstrual dysphoric disorders, negative symptoms in schizophrenia, or impulsive aggression. These might be related to the effects of levels of allopregnanolone on the modulation of GABAA receptor function^13^,^58^,^59^. The evidences from the preclinical and clinical studies suggested that GABA played a role in the pathophysiology of the diabetic-related depression^60^. The study has shown that the GABAA agonist modulator interacted on the brain by changing the expression of α2 GABAA receptor subunit to elicit the neuroprotective effects on depressive-like behavior with diabetes mellitus^61^. The protective effects of allopregnanolone have been also reported in animal models of peripheral diabetic neuropathy (i.e., rats rendered diabetic by STZ injection). The neuroactive steroid improves sciatic nerve conduction velocity, mRNA levels of a myelin protein, such as the peripheral myelin protein 22, thermal threshold, skin innervation density^57^. It is also worth noting that allopregnanolone produces the antidepressant-like effects in T2DM.

On this point of view, it has demonstrated that the decreased levels of allopregnanolone were reversed by Flu (10.8 mg/kg, i.p) and MF. This finding was supported by that SSRIs were able to reverse the decreased allopregnanolone^16^. Also, clinical studies demonstrated that treatment with SSRIs normalized the allopregnanolone content of CSF in patients with depression^62^. The observation obtained in the present study indicating that similar to Flu (10.8 mg/kg, i.p) and MF, AC-5216 stimulated the lowered levels of allopregnanolone biosynthesis in the prefrontal cortex (Fig. 7A) and hippocampus (Fig. 7B). AC-5216 may represent an interesting therapeutic perspective based on the explanation that these ligands are able to increase the biosynthesis of neuroactive steroids^56^. This is consistent with a previous report that anxiolytic-like effects of AC-5216 were associated with the increased levels of allopregnanolone in the brain^49^. It is interesting to note that other TSPO ligands, such as Ro5-4864 and etifoxine, exerted the neuroprotective effects in various animal models of neurodegeneration by allopregnanolone biosynthesis^23^,^63^. However, the increased levels of allopregnanolone by AC-5216 (Fig. 7D,E) were blocked by PK11195, suggesting that the antidepressant-like effects of AC-5216 in T2DM animal model were associated with allopregnanolone biosynthesis, which was mediated via TSPO in the brain. These findings were also consistent with our previous studies showing that the anti-PTSD-like effects of AC-5216 were mediated by TSPO and allopregnanolone biosynthesis^37^,^40^. More researches have shown that TSPO activation was effective in ameliorating the severity of diabetic neuropathy through a local increase of neuroactive steroid levels^23^. We also found that the lower allopregnanolone biosynthesis in serum (Fig. 7C) was not reversed by AC-5216 indicating that action of AC-5216 on depression in T2DM was associated with the stimulation of allopregnanolone biosynthesis in the CNS.

Interesting in our present study, we found that the effects of Flu (10.8 mg/kg, i.p) and MF were similar to Met (1.8 mg/kg, i.p) on levels of PG (Fig. 6A), TC (Fig. 6B). TG (Fig. 6C) and INS (Fig. 6D). Our findings were supported by the previous study that Flu induced lowered blood glucose and in patients with diabetes^64^. Also, Flu enhanced the insulin sensitivity in obese patients and ameliorated the function of pancreatic β-cell secretory in drug-naive major depressive disorder (MDD) patients^65^,^66^. In addition, when it came to TC and TG, data showed that both of the parameters were decreased by chronic Flu treatment in patients with MDD^67^. Partially in line with Met (1.8 mg/kg, i.p), Flu (10.8 mg/kg, i.p) and MF, HFD-STZ-induced effects on PG (Fig. 6A) and INS (Fig. 6D) were reversed by AC-5216 without affecting TC (Fig. 6B) and TG (Fig. 6C). The results indicated that the antidepressant-like effects of AC-5216 were partially associated with the alternation of PG and INS and in agreement with that administration of TSPO ligands to obese mice reduced weight gain and lowered glucose level^68^. The possible mechanism is involved in activation of TSPO that improves glucose uptake. The previous study showed that the presence of TSPO by itself might provide the protection for the normal cellular activities of adipocytes^68^. Knockdown of TSPO in newly differentiated healthy adipocytes led to negative metabolic consequences, including the reduction of insulin stimulated glucose uptake and irregularities in adipokine release. Although many details remain to be elucidated, the data presented together have shown that activation of TSPO by ligands, with the ingestion of excess nutrition, may promote lipid consumption and improve adipocyte function, insulin sensitivity and glucose homeostasis, thus serving as therapeutic strategies to cure obesity and T2DM^68^. Further studies should be conducted to investigate the putative mechanisms of the interaction on depression in T2DM (e.g allopregnanolone biosynthesis, GABA system), as well as the role of TSPO in the modulation of T2DM aiming at new strategies to treat depression in patients with T2DM.

## Conclusion

The present study demonstrated that the role of TSPO and allopregnanolone in the treatment of depression in T2DM. These effects were associated with the allopregnanolone biosynthesis that was mediated by TSPO. The findings advance our knowledge that TSPO selective ligands may be a promising new pharmacological class of drugs for the future treatment of depression in T2DM.

## Additional Information

**How to cite this article**: Qiu, Z.-K. *et al.* The antidepressant-like activity of AC-5216, a ligand for 18KDa translocator protein (TSPO), in an animal model of diabetes mellitus. *Sci. Rep.* **6**, 37345; doi: 10.1038/srep37345 (2016).

**Publisher’s note:** Springer Nature remains neutral with regard to jurisdictional claims in published maps and institutional affiliations.
